# Primary malignant multiple osteogenic sarcomas: a rare clinical image

**DOI:** 10.11604/pamj.2022.42.263.35710

**Published:** 2022-08-10

**Authors:** Sonu Rajwade, Shweta Parwe

**Affiliations:** 1 Department of Panchakarma, Mahatma Gandhi Ayurveda College Hospital and Research Centre, Datta Meghe Institute of Medical Sciences, Wardha, India

**Keywords:** Physical examination, metaphysis, knee joint

## Image in medicine

We present the case of a 12-year-old male patient, who came to the emergency department with a complaint of pain in bilateral knee joints, pain in bilateral lower legs, multiple swelling over the bilateral knee joint and in the leg region, difficulty in walking, stiffness in the bilateral knee joint since he was one year. There is no medical significant history regarding the disease condition. On physical examination, the swelling was in the region of the metaphysic of the distal end of the bilateral femur and proximal end of the bilateral tibia, skin over the swelling was shiny with tenderness and margins were irregular, on the biopsy and X-ray findings patient was diagnosed as primary malignant multiple osteogenic sarcomas. The patient was referred to the dermatology department for further management.

**Figure 1:**
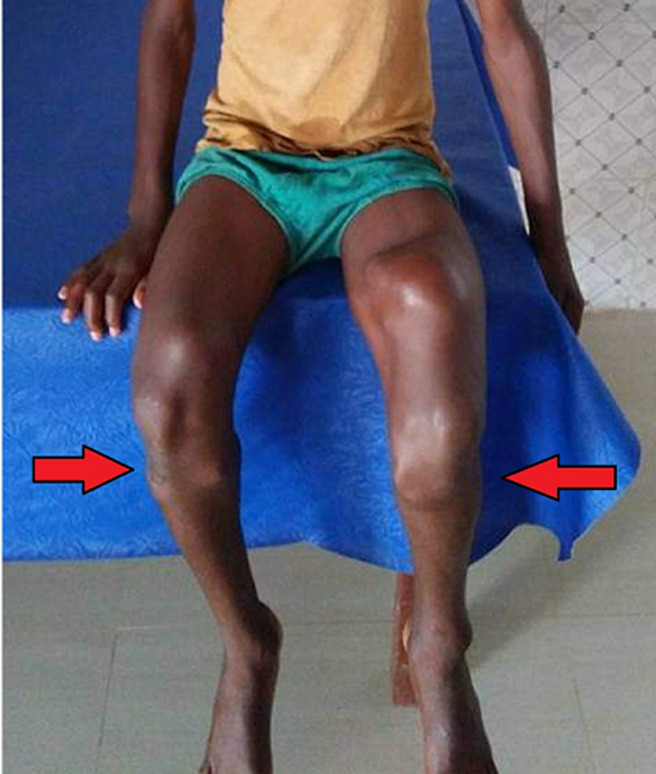
primary malignant multiple osteogenic sarcoma of lower limbs

